# Medical–Surgical Implications of Branching Variation of Human Aortic Arch Known as Bovine Aortic Arch (BAA)

**DOI:** 10.3390/jpm14070678

**Published:** 2024-06-24

**Authors:** Andreea Rotundu, Alin Horatiu Nedelcu, Razvan Tudor Tepordei, Marius Constantin Moraru, Dragos Andrei Chiran, Andra Oancea, Alexandra Maștaleru, Alexandru-Dan Costache, Costin Chirica, Cristina Grosu, Florin Mitu, Maria Magdalena Leon

**Affiliations:** 1 Doctoral School, “Grigore T. Popa” University of Medicine and Pharmacy, 16 Universității Street, 700115 Iasi, Romania; rotundu.andreea@d.umfiasi.ro (A.R.); chirica.costin@d.umfiasi.ro (C.C.); 2 Department of Medical Specialties I, “Grigore T. Popa” University of Medicine and Pharmacy, 700115 Iasi, Romania; andra.oancea@umfiasi.ro (A.O.); alexandra.mastaleru@umfiasi.ro (A.M.); dan-alexandru.costache@umfiasi.ro (A.-D.C.); florin.mitu@umfiasi.ro (F.M.); maria.leon@umfiasi.ro (M.M.L.); 3 Department of Morpho-Functional Science I, Discipline of Anatomy, “Grigore T. Popa” University of Medicine and Pharmacy, 16 Universitatii Street, 700115 Iasi, Romania; razvan.tepordei@umfiasi.ro (R.T.T.); marius.moraru@umfiasi.ro (M.C.M.); dragos-andrei.chiran@umfiasi.ro (D.A.C.); 4 Radiology Clinic, Clinical Rehabilitation Hospital, 700661 Iasi, Romania; 5 Clinical Rehabilitation Hospital, 700661 Iasi, Romania; 6 Department of Neurology, “Grigore T. Popa” University of Medicine and Pharmacy, 700115 Iasi, Romania; cristina.grosu@umfiasi.ro; 7 Department of Neurology, Rehabilitation Hospital, 700661 Iasi, Romania

**Keywords:** bovine aortic arch, prevalence, imaging investigations, coarctation, thoracic aortic disease, stroke, endovascular interventional treatment

## Abstract

(1) Background: The aortic arch (AA) branching model is challenging, considering the multiple anatomical variations documented in existing research. The bovine aortic arch (BAA) is the most prevalent anatomical variation among these. This variant of AA branching has long been considered a nonsymptomatic malformation, having been discovered incidentally during imaging investigations for other causes. However, more recent studies have demonstrated that BAA shows a frequent association with coarctation of the aorta (CoA), thoracic aortic disease (TAD), and stroke. At the same time, given the current context of increasing activity in the fields of interventional and surgical procedures in the aorta and its branches, it is very important to know the medical–surgical implications of this anatomical variant. (2) Methods: We conducted a comprehensive review using PubMed and Embase, focusing specifically on randomized trials and cohort analyses that examined the medical–surgical implications of BAA. We assessed information related to studied groups, medical procedures, and study outcomes. Initially, we identified 8454 studies, and after rigorous evaluation, we narrowed down our review to 25 articles. (3) Discussions: The intervention consisted of assessing the risks associated with BAA through different imaging investigation methods such as computer tomographic angiography (CTA), magnetic resonance imaging (MRI), or ultrasonography (US). The following results were evaluated: the prevalence of the BAA, the importance of imaging investigations in establishing the diagnosis and the therapeutic management and monitoring the evolution of patients with the BAA, the association of the BAA with CoA, TAD, and stroke, and the potential risks of interventional treatment in patients with the BAA. (4) Conclusions: The prevalence of the BAA differs both between different ethnic groups and between genders. Advanced imaging methods such as CTA and 4D flow MRI allow detailed descriptions of supra-aortic vascular anatomy and information about blood flow velocities, direction, and turbulence in the AA. US remains an easy and valuable imaging investigation, with the potential to detect and correctly diagnose the BAA and its hemodynamic implications. Anatomical variations in the AA are associated with increased rates of TAD, CoA, and stroke, necessitating early diagnosis and increased supervision of patients with such incidentally observed abnormalities. In addition, there is a need to further develop and refine the surgical techniques used and personalize them to the individual characteristics of patients with the BAA.

## 1. Introduction

There is a wide spectrum of aortic arch (AA) branching anomalies, some of which are clinically nonsymptomatic, and discovered incidentally during investigations for other pathologies. Others are clinically manifested by symptoms such as severe dyspnea associated with respiratory distress or “dysphagia lusoria” caused by esophageal compression [1]. In some cases, anatomical variations of the AA may occur alongside congenital cardiac anomalies or chromosomal genetic abnormalities [2,3]. Thus, precise knowledge of the AA’s genesis is essential to systematize and analyze the multitude of malformations of this anatomical structure.

The development process of the large vessel system lasts from the third to the eighth week of gestation. [4]. Bae et al. [5] proposed two distinct models of embryogenesis that can explain this system’s forms.

According to the Rathke diagram, the large vessel system derives from six pairs of arteries in the pharyngeal arch. The six pairs of arches are known to connect the dorsal and ventral primitive aortas. These primitive arches undergo processes of regression and remodeling to form structures of the large vessel system subsequently. After they regress, the remaining portions of the first and second arches become the maxillary, hyoid, and stapedii arteries. The third arch participates in the formation of the common carotid arteries and the proximal internal carotid arteries. The definitive AA is derived from the primitive fourth arch. In most cases, the fifth arch regresses completely, but rarely may it form the double-barrel aorta. The sixth arch becomes part of the pulmonary arteries and ductus arteriosus [6,7].

Another model that explains AA embryology is the theoretical concept of the double arc described by Edwards. It involves paired aortic arches and ductus arteriosus arranged on either side of a single dorsal aorta. The anteriorly located carotid arteries and the posteriorly located subclavian arteries branch off from the AA. The left AA forms because of a regression process of the right aortic arch, right lateral canal, and right dorsal aorta. The left dorsal aorta forms the descending part of the thoracic aorta and the distal part of the AA. The first portion of the right dorsal aorta gives rise to a segment of the right subclavian artery [8,9].

The different AA malformations may be related to deviations occurring at different stages of the double AA formation process proposed by Edwards. When certain segments persist or regress abnormally, it leads to the development of certain anomalies in the AA.

**Figure 1:**
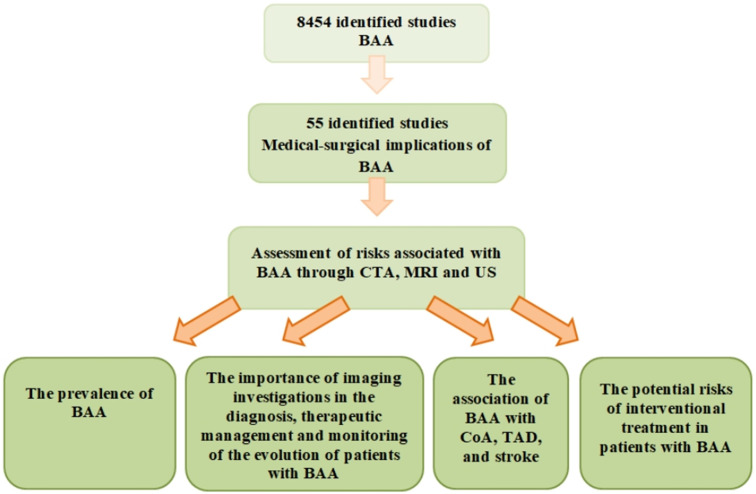
Literature search strategy.

The typical order of branching of the AA begins, from right to left, with the brachiocephalic trunk (BT) and then continues with the left common carotid artery (LCCA) and the left subclavian artery (LSA). The BT, in turn, divides into the right common carotid artery (RCCA) and the right subclavian artery (RSA) (Figure 1). A special component, both in functional importance and due to the great variability of origin, consists of the vertebral arteries.

In most cases, these arteries branch into the subclavian arteries, but sometimes they can derive directly from the AA or the common carotid arteries [7].

**Figure 2:**
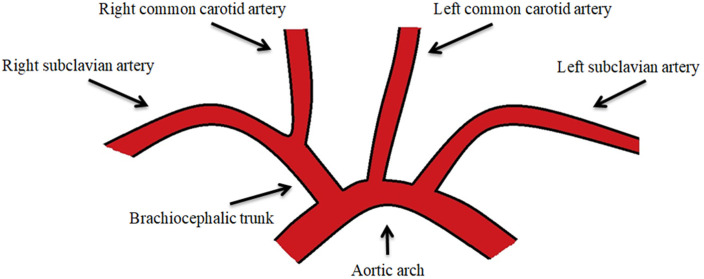
The normal branching pattern of the aortic arch.

**Figure 3:**
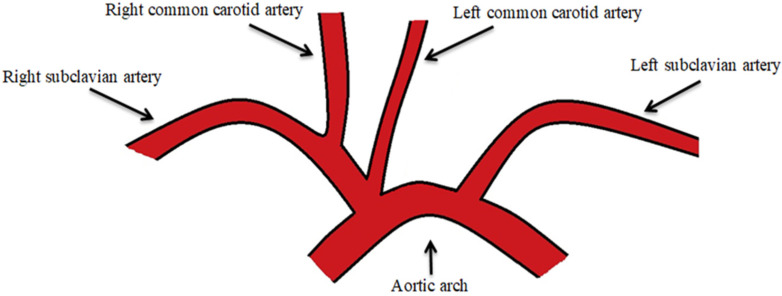
The Type I bovine aortic arch.

**Figure 4:**
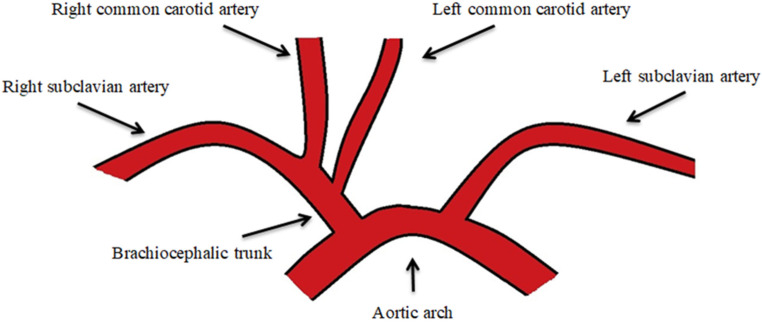
The Type II bovine aortic arch.

The most common branching variation of the left AA is the bovine aortic arch (BAA), with a prevalence ranging from 7% to 25% [10,11,12,13]. In turn, this anatomical anomaly has two branching patterns: the Type I bovine aortic arch (T1BA) in which the BT and LCCA share a common origin at the AA (Figure 2), and the Type II bovine aortic arch (T2BA) in which the LCCA originates directly from the BT (Figure 3) [14]. The difference between T1BA and T2BA prevalence may be the result of complex processes of embryological development and gene expression that determine the organization and formation of the aortic arch during intrauterine life. In the T1BA, BT (which splits into the RCCA and RSA) and LCCA share a common origin in the AA, this variant being the result of a more simplistic developmental anomaly. This is why the T1BA is often considered developmentally closer to the normal anatomy of the AA. The T2BA results from more complex anomalies in aortic arch formation, and this variant is characterized by the LCCA arising directly from the BT. At the same time, certain genetic variants or regulatory factors in AA development may favor one of the anatomical configurations, leading to a higher prevalence of one over the other (Figure 4).

A morphological association frequently encountered in clinical practice, asymptomatic in many cases, is the origin of aberrant RSA. This specific condition occurs when the right AA involutes between the RSA, the RCCA, and the regression of the right ductus arteriosus. In this anomaly, the proximal part of the aberrant RSA originates in the lower part of the right dorsal aorta instead of the usual location, the right fourth arch [5,15]. It passes posterior to the esophagus, which can lead to a swallowing difficulty known as “dysphagia lusoria”. It is essential to check for the presence of the Kommerell diverticulum, as its association, especially if it is enlarged or forms an aneurysm, signifies a higher risk [16].

Although the BAA has long been considered an asymptomatic anatomical variant of aortic arch branching, the latest research findings suggest that the BAA shows a frequent association with coarctation of the aorta (CoA), thoracic aortic disease (TAD), and stroke. In addition, according to results reported in several studies, people with this anomaly have an increased risk of morbidity associated with endovascular and surgical procedures performed on the AA and its branches. Given these findings, healthcare professionals need to know the medical and surgical implications of the BAA so that they can opt for personalized therapeutic management for this category of patients to minimize the risks associated with this anomaly.

## 2. Materials and Methods

In this review, we conducted an analysis of the medical and surgical implications of BAA. We searched for relevant articles in the Embase and PubMed databases using subject headings and keywords. Imaging methods such as CTA, MRI, and US were used to study the prevalence of BAA, its relationship with CoA, TAD, and stroke, and the potential risks associated with interventional treatment in patients with BAA. The importance of these imaging techniques in diagnosing BAA, selecting appropriate treatment, and monitoring outcomes was also assessed. Initially, we focused on studies published in the last decade (2014–2024) and selected 25 articles out of a total of 8654 after carefully reviewing titles, abstracts, and full texts (Table 1).

## 3. Results

### 3.1. The Prevalence of the Bovine Aortic Arch in Populations from Different Geographical Regions

In their study on a population of 90 patients, Ahn et al. [17] compared the incidence of the BAA with that reported in previous research. In terms of ethnicity, the majority of the patients were Caucasian, with a smaller number of Hispanic, Asian, African American, Indian, and other nationalities. The overall incidence of the BAA in the study group was significantly higher (35.16%) compared to previous reports (7–25%) [10,11,12,13]. In addition, Caucasians had a lower incidence of the BAA compared to other ethnicities, while Hispanics had almost twice the prevalence of the BAA compared to Caucasians (50% vs. 27.78%). The prevalence of the BAA was 40.0% in women (n = 60) and 26.67% in men.

Moorehead et al. [10] investigated the presence of various types of bovine arch in a group of 817 individuals from the USA. The participants comprised 47% Caucasian and 50% African American individuals. Results showed that 31.1% of the participants had a BAA, with 14.9% having T1BA and 16.2% having T2BA. The prevalence of T1BA was higher than T2BA, which aligned with earlier research conducted by Ahn et al. [17].

In a study involving 444 South American subjects conducted by Prada G. et al. [11], the prevalence of anatomical variations of AA was found to be 40.1%, higher than the previously reported range of 7–25% [10,11,12,13]. The most common variation was T1BA (type 2)—27.9%, followed by T2BA (type 3)—9.9%, results similar to previous research. Another notable finding was that the prevalence of global anatomic variations was higher in females than males (42.3% vs. 35.9%), reinforcing the findings of the previously mentioned study [17].

Regarding the prevalence of the BAA in a study of 2037 patients in Turkey, it was 15.5% (n = 315), which was the most common abnormality of AA in this group as well. The BAA was also statistically significantly more frequent in female than in male subjects (18.2% versus 12.8%) [18].

Another study, conducted on a group of 1000 patients of Turkish origin, revealed a 14.1% incidence of bovine arch [13], which is similar to that reported in the previous study conducted in the same geographical region [18]. The BAA was also the most frequent aortic arch anomaly and was identified with a higher prevalence in females than in males (22.1% versus 20%).

Our review showed that the prevalence of the BAA varies between different geographical areas. For example, the study by Prada et al. [11] in South America found a higher prevalence of anatomical variations of the AA, which could be attributed, on the one hand, to the lack of such previous research in this region. When studies are conducted on certain previously less researched ethnic groups, it is possible to discover anatomical features and variations that have not been commonly identified elsewhere.

The difference in the prevalence of the BAA in the study by Ahn et al. [17], compared to that reported in other research, could be attributed to the significant ethnic diversity of the study population, which included a wide range of ethnicities. It is well known that in South America, the population also presents a complex history of genetic admixture between Native Americans, descendants of European colonizers, Africans, and other ethnic groups that migrated to these territories, making the group studied by Prada et al. [11] racially mixed. Genetic and historical diversity can lead to a greater variety of anatomical features and physiological variations, even in the structure of AA. Certain genetic characteristics specific to Caucasians may make them less likely to develop the BAA compared to other ethnicities, which explains the approximately two-fold lower prevalence of this anatomical abnormality in Caucasians compared to Hispanics (27.78% vs. 50%) reported in the study by Ahn et al. [17].

In addition, environmental factors may interact with genetic factors to influence cardiovascular development. Certain environmental factors, such as nutrition, exposure to toxins, or environmental conditions, may have a different impact on the Hispanic population than Caucasians, which could contribute to the observed differences in the prevalence of the BAA.

Finally, yet importantly, the lower socio-economic level of part of the South American population may have contributed to a more limited access to healthcare services. Lack of adequate medical infrastructure and access to specialized medical services could have been implicated in the underdiagnoses and underreporting of anatomical variations of AA by the time of the study we included in this analysis.

On the other hand, the similar prevalence of the BAA in studies conducted in Turkey by Karacan et al. [13] and by Terzioğlu et al. [18] to that generally reported in the literature can be explained by the fact that this region is genetically and ethnically homogeneous with a predominantly Turkish population. Another thing that could contribute to reporting a similar prevalence of this anatomical anomaly could be the use of the same criteria and protocols for BAA diagnosis as in other international studies. Also, easy access to international research and data in the literature allow specialists in this country to keep abreast of findings and information about anatomical variations such as the BAA.

Our study identifies a different prevalence of BAAs depending on the geographical area and population type. A higher incidence was demonstrated in the Hispanic population, followed by Caucasians and the population from Asia Minor. However, there are some limitations determined by the addressability of the population in underdeveloped or developing countries to imaging investigations such as angio CT and MRI.

### 3.2. The Importance of Imaging Investigations in the Diagnosis, Treatment, and Follow-Up of Patients with This Anatomical Variant

Computed tomography (CT) is an essential tool for diagnosing and assessing AA abnormalities. Traditional catheter angiography is not ideal for describing AA morphology due to overlapping vascular branches. The advent of multidetector computed tomographic angiography (CTA) has made it the main imaging method for evaluating the aorta and its branches, allowing high-resolution images to be obtained in a short time [13,17,18,39,40].

In his study, Prada G. et al. [11] evaluated the CTA of 444 patients to establish a new classification for AA branching patterns and the link between certain anatomical variations and aortic pathologies. 460 CTAs with 3D reconstruction were reviewed. The authors classified the AA into four types, including its normal anatomy—type 1, which had the highest prevalence in the study group (59.9%), followed by T1BA—type 2 (27.9%), T2BA—type 3 (9.9%) and left vertebral artery originates from the aortic arch—type 4, the latter having the lowest prevalence (2.2%). The prevalence of aortic pathology diagnosis among people with branching variations of AA was 14%, distributed by branching type as follows: type 2 14.5%, type 3 11.4%, and type 4 20%.

In the study by Karacan A et al. [13], conducted on a group of 1000 patients, the authors analyzed how often variants in AA branching pattern occurred with the use of 64-section multidetector CTA. They identified seven types of anatomical variations in the AA, with 79.2% of patients showing a normal branching pattern. Anatomical variations of the AA were found in 20.8% of patients, the most frequent being T1BA (14.1%). This retrospective, retrospective, cohort, large-scale study, like the one by Prada et al. [11], suggests that CTA is a valuable investigation that allows the accurate examination of supra-aortic vessels due to the superior quality of spatial detail and the possibility of providing images in multiple planes and three dimensions.

Although CTA provides important details about the AA branching model, it comes with some drawbacks, such as exposure to ionizing radiation and the use of iodine-based contrast agents. Magnetic resonance angiography is another imaging technique, devoid of ionizing radiation, that can provide additional details about the differences between the standard AA and BAA in terms of hemodynamic flow characteristics and regional shear localized shear forces on vascular endothelium.

In a study based on four-dimensional flow magnetic resonance imaging (4D-flow MRI), hemodynamic constants were compared for three variations of AA. The researchers aimed to explain how a specific variation in AA anatomy might contribute to the development of type B aortic dissection (TBAD). The study participants were divided into two groups, with TBAD (n = 185) and without TBAD (n = 367). Using 4D-flow MRI, the researchers concluded that the inner curvature of the BAA experienced greater systolic shear stress compared to both normal AA and AA with aberrant RSA [19].

Clerici et al. [20] performed a study based on ultrasonography explorations (US) to determine the prevalence of the BAA in fetuses in a group of 742 women at 21–39 weeks gestation. In addition, the scientists tried to identify any hemodynamic differences between patients with the standard AA and those with the BAA by assessing epi-aortic and middle cerebral artery (MCA) blood flow by Doppler ultrasound. Finally, they examined neonatal outcomes in both cohorts to elucidate the clinical significance of the BAA branching model compared to the conventional branching pattern. Of the patients studied, 6.06% were found to have the BAA, with hemodynamic assessments performed on thirty-nine fetuses (thirty-three with a normal AA model and six with the BAA). In the BAA group, the researchers observed a rapid monophasic systolic wave with a rapid peak systolic velocity (time to peak systolic velocity (TPV)—low) along with minimal diastolic flow. Although both types of AA showed similar flow patterns, significant hemodynamic variations were found between the normal AA group and the BAA group. On the other hand, MCA flows did not register statistically significant differences between the two groups studied. Previous studies suggest that BAA patients may be at higher risk of thoracic aortic aneurysm (TAA) and thoracic aortic dissection (AD), highlighting the importance of assessing hemodynamic constants in individuals with this anatomical variant of AA [25,41].

Turek et al. [21] focused on determining the accuracy of bovine aortic arch (BAA) identification using cardiac ultrasonography. They compared the results from the patients’ echocardiograms performed by a cardiologist with the results obtained from the echocardiograms performed before surgery. Surprisingly, pre-surgical findings detected the BAA in only 6.1% of patients, whereas subsequent examination by a cardiologist classified 28.6% of the arches as having bovine anatomy. This suggests that the presence of bovine arch anatomy is frequently missed in preoperative assessments, emphasizing the importance of careful and accurate interpretation of echocardiographic images.

In the diagnosis of the bovine aortic arch, imaging investigations play a primary role. Among these useful investigations are ultrasonography of the aorta and MRI, but multidetector computed tomographic angiography represents the “gold standard” due to the morphological details, the short duration of the exploration, and the accessibility. Moreover, most of the anatomical variations of the aortic arch are asymptomatic and can be detected accidentally during chest CT.

### 3.3. The Association of the Bovine Aortic Arch with Aortic Coarctation, Thoracic Aortic Disease, and Stroke

#### 3.3.1. Coarctation of the Aorta (CoA)

A study reviewed CTAs from a cohort of children with congenital cardiovascular anomalies (n = 700) aged 2 days to 18 years to investigate the occurrence of the BAA in patients with CoA. Of the participants, 16.71% had CoA. The study found that the BAA was more prevalent in patients with CoA (5.98%) compared to those without CoA (2.06%), demonstrating a significant association between the CoA and BAA [22].

Turek JW et al. [21] conducted a study using echocardiography aimed at assessing the risk of aortic recoarctation (ReCoA) in patients with the BAA after the left thoracotomy approach for extended end-to-end CoA correction. The scientists also attempted to identify an anatomical cause for ReCoA. This hemodynamic condition associated with ReCoA was found in 5.7% of patients with normal AA anatomy and 28.6% of those with bovine arch anatomy. The anastomosis index was also calculated for cases with angiographic imaging, revealing that anastomotic length was markedly shorter in BAA patients. Thus, the pediatric population with BAA anatomy undergoing extensive CoA repair faces an increased risk of recurrent arch obstruction attributable to the reduced anastomotic length achieved during surgery.

Meyer et al.’s [23] research on 178 infants aimed to identify the cause for the higher rates of ReCoA in bovine arches observed by Turek et al. [21] in the above study. They also aimed to assess the branching of vessels in the bovine arch in contrast to the standard AA, to understand their embryological origins, and to determine the nature of AA branch displacement. The findings showed that the BAA differs from normal AA in branching morphology, with shorter distances between certain artery segments. In addition, it was demonstrated that the short length of the vessel available for coarctation repair through the left thoracic approach specific to the BAA anatomy is associated with an increase in recurrent CoA in this category of patients, as previously reported by Turek et al. [21].

Froud et al. [24] had as the main aim of their study to evaluate the length of vessels available for anastomosis in patients with the BAA. This abnormality was seen on CT scans in 34% of the 169 infants in whom CoA was corrected through a left thoracic approach. The study showed that infants with the BAA had significantly shorter anastomotic distances compared with those with normal AA anatomy. Therefore, this study supports the conclusions of the above study [23], suggesting that the shorter vessel length available for anastomosis in patients with the BAA may affect surgical outcomes and increase ReCoA rates.

There is a bidirectional correlation between coarctation of the aorta and branching variations of the aortic arch, especially of the bovine aortic arch. In addition, the presence of the BAA is associated with a predisposition to recurrence of the aorta coarctation after surgical treatment.

#### 3.3.2. Thoracic Aortic Disease (TAD)

The pathophysiological process underlying the association of the BAA with thoracic aortic disease is not fully elucidated. Pham et al. [42] observed that in individuals with TAD and bovine arch, the middle aortic tunica is thinner, while the internal aortic tunica is thicker, in contrast to those with the BAA but without thoracic aortic pathology. A thicker tunica intima was associated with larger-diameter aneurysms, while a thinner tunica media was associated with smaller aneurysms. Changes in the shape of the aortic arch can affect blood flow patterns. In the BAA, the flow and direction of blood are considerably altered. This alteration, combined with fewer vessels branching directly from the aortic arch, can increase the velocity of blood flow through the aorta, leading to greater pressure on the vessel wall and contributing to aortic damage and dilatation or rupture [41]. Computer simulations of blood circulation and imaging techniques such as magnetic resonance imaging have previously been used to examine circulatory dynamics in different AA branching patterns [43,44]. These tools could also be beneficial in assessing unique hemodynamic features in patients with conditions such as the BAA and TAD.

The study led by Dumfarth et al. [25] investigated the potential of AA variants as markers for TAD. The authors assessed the presence of aortic arch variants on imaging investigations of 556 patients undergoing surgery for TAD. Their demographics were compared with a cohort of 4617 subjects without known TAD. The researchers found a significantly higher number of AA abnormalities in the TAD group compared to the control group (33.5% vs. 18.2%). The BAA was the most common abnormal branching pattern of AA in patients with TAD, with a prevalence of 24.6%. Patients with TAD and AA branching variations were younger, underwent AA surgery more frequently, and had lower rates of high values of blood pressure, as well as higher rates of aortic bicuspids compared with TAD patients with normal AA.

In contrast to the patients included in the group studied by Dumfarth et al. [25], the 156 patients included in the second part of the study by Moorehead et al. [10] were advanced age, exhibited a higher incidence of hypertension, hyperlipidemia, and calcification of the aortic valve. Of the 156 patients with TAD, 26 had aortic dissection (AD), and 130 had thoracic aortic aneurism (TAA). The study also found that the BAA was more prevalent in patients with AD compared to healthy individuals. This indicates that the BAA is more frequently discovered in people with AD, suggesting a higher risk of thoracic aortic disease and emphasizing the need for careful clinical surveillance.

Sun et al. [26] investigated the link between the BAA and bicuspid aortic valve and their association with an increased risk of TAD. In their study involving 449 patients, 21.2% had a BAA, representing almost 80% of all anatomical variants. They found that patients with a BAA had a higher prevalence of aortic bicuspids compared to those with normal AA. However, the study concluded that although the BAA was linked to aortic bicuspids, the bovine arch was not independently associated with a heightened susceptibility to TAD. Instead, aortic bicuspids and masculine gender were identified as risk factors for TAA.

A group of researchers retrospectively analyzed CT scans of 21,336 patients aged 50 years and older (50–85 years). Six hundred-three patients (2.8%) with AA abnormalities were identified in the study group. The most common abnormality identified was the BAA (354 patients, 58.7%). The prevalence of TAA was significantly increased in the cohort with AA abnormalities compared to the group with standard AA (65 patients, 10.8%, compared to 844 patients, 4.1%). Factors linked to an increased risk of TAA comprised anomalies in the AA, disease of the aortic valve, male gender, and high blood pressure values. The study found that about 3% of older adults have AA abnormalities, with a significant occurrence of TAA in this group. This highlights the importance of implementing a specific surveillance strategy for TAA in older adults [27].

Given that TAA is a pathology with familial aggregation in some cases, it has been hypothesized that the BAA also originates from a genetic defect, thus indicating the heritability of this anatomical variation. Considering that TAA associated with the BAA increases the risk of mortality, understanding the inheritance of the BAA is essential not only from a theoretical point of view but also to direct the monitoring of relatives of the BAA subjects. To investigate the heritability of the BAA, a study involving 24 patients with the BAA and TAA examined the prevalence of AA configuration in their primary and secondary family members. Of 43 relatives with available imaging scans, 53% had a BAA, suggesting a strong genetic component with an estimated heritability of 0.71 [28].

Another study in a Japanese population examined the prevalence of AA branching pattern diversity in these individuals, comparing patients with TAD to healthy controls. The study group (group A) consisted of 815 Japanese patients with TAD, defined as TAA (diameter ≥ 45 mm) and AD, who had undergone AA surgery. The control group (group C) consisted of 1506 patients who had undergone trauma (group C). The two groups of patients had undergone preoperative CT and CT screening for trauma, respectively. The scientists found that AA branching abnormalities were present in 17.2% of group A and 14.7% of group C. Patients with TAA from group A had significantly more AA abnormalities compared to group C, including a BAA and aberrant RSA. Regarding aneurysm location, the proximal aneurysm was detected more frequently in patients with a BAA (15.2%), and the distal aneurysm was detected more frequently in patients with aberrant subclavian artery (3.7%). Therefore, the study concluded that BAAs could be a risk factor for TAAs, especially those located in the proximal segment [29].

Dumfarth et al. [30] found in their study that the BAA is a risk factor for specific entry sites in acute DeBakey type I aortic dissection (AADA). The study involved 315 patients undergoing AADA surgery, divided into cohorts of patients: those with a BAA (n = 49) and those without a BAA (n = 264). The BAA had a prevalence of 15.6% in patients with AADA. The BAA was present in 15.6% of AADA cases. In addition, the site of entry location was more common in the AA among patients with a BAA compared to those without a BAA. The BAA and preoperative competent aortic valve were independent predictors of aortic arch dissection. A similar result was observed in a 2014 study of 157 patients by Dumfarth et al. [31], in which patients with a BAA had a significantly higher rate of primary rupture location in the AA compared with those without a BAA.

We can conclude that patients with a BAA present an alteration of the blood flow at the level of the aortic arch and the thoracic aorta. The morphological parietal changes determined by the flow turbulences are at the origin of the thoracic aortic disease, especially the aneurysm and dissection of the aorta.

#### 3.3.3. Stroke

One study investigated the association between embolic strokes and the BAA to determine whether this anatomical abnormality may become a possible biomarker for determining the risk of developing stroke. The study group consisted of 152 subjects presenting acute embolic stroke in the anterior territory of the cerebral vasculature in their background. The control group consisted of 322 patients with no history of embolic stroke. Head and neck CTA investigated both groups. The BAA was more frequent in the group of patients with ischemic stroke due to the embolism (25.7%) compared to those without this pathology in the antecedents (17.1%). T1BA was identified approximately equally frequently in both groups (15.1% vs. 12.1%). T2BA was identified more often among patients with stroke due to the embolic cause compared to those without a history of stroke (10.5% vs. 5.0%) [32].

Several previous studies have shown that ischemic strokes in the left hemisphere are generally more common and are often associated with a poorer prognosis than those in the right hemisphere [45,46]. However, data in the literature on the tendency to localize right or left-sided cardio-embolic strokes are conflicting [46,47].

Gold et al. [33] investigated whether there is a preference for the location of strokes of embolic cause in one of the two cerebral hemispheres, comparing a group of patients with a BAA with a group of subjects with normal configuration AA. The study involved 119 patients with acute strokes in the anterior cerebral vascular territory and identified that 33% had a BAA. The main causes of embolic stroke were atrial fibrillation in 67% of cases and congestive heart failure with ejection fraction <30% in 15% of cases. In addition, patients with BAAs showed an equal chance of stroke in both hemispheres, while those with standard AA tended to have strokes more often in the right hemisphere, although this trend was not statistically significant. Therefore, the study suggests that BAAs may be an independent risk factor for stroke of embolic cause.

Another study, conducted on a group of 615 known subjects with atrial fibrillation who also suffered embolic strokes, investigated whether AA branching variations influence the laterality of cardioembolic stroke. All patients had imaging investigations at the time of stroke (CT—32% or MRI—68%). Among patients with normal AA (n = 424), stroke distribution was in the left cerebral hemisphere at 43.6%, in the right cerebral hemisphere at 45.1%, and in both cerebral hemispheres in 11.3% of cases. In the BAA group (n = 191), the distribution of stroke was left in 51.3%, right in 35.6%, and bilateral in 13.1% of cases. The BAA was more commonly associated with left cardioembolic stroke compared to standard arches. The central observation of this study was that subjects with a bovine aortic configuration have a statistically significantly higher propensity for left-sided cardioembolic stroke compared to those with standard AA [34].

Dumfarth et al. [30] examined patients with bovine arch are at higher risk of experiencing postoperative neurological complications following acute type A aortic dissection (AADA) repair. 12.4% of all patients (n = 39) suffered a stroke. Patients with BAAs more frequently had a stroke (24.5%) compared to those with normal anatomy (10.2%). Thus, the presence of BAAs independently contributed to stroke risk after surgical treatment of AADA.

An observational study, which included 200 patients, had as its main objectives to determine the prevalence of different anatomical features of AA in participants who suffered ischemic stroke and to assess the effect of aortic morphology on early-onset stroke. The normal AA configuration was defined as a type I aortic arch, which was subclassified into three types based on the distance between the origin of the LV and the apex of the AA. Subtype 2 (distance mentioned is between 1 and 2 LCCA diameters) was associated with the earliest onset of stroke in individuals with normal AA. The bovine arch was, in turn, subclassified into two types, type A (equivalent to T2BA) and type B (equivalent to T1BA), correlating with the youngest age at stroke presentation [35].

In our conception, the common origin of the brachiocephalic arterial trunk and the left common carotid artery determines major turbulent blood flow. More precisely, these turbulences determine the early appearance and accelerated evolution of atherosclerotic changes in the left carotid system with the subsequent installation of ischemic stroke. In conclusion, BAAs can be considered an etiological factor of ischemic stroke, a fact demonstrated by its location predominantly in the left hemisphere.

### 3.4. Potential Risks Associated with Endovascular Interventional Treatment in Patients with Bovine Aortic Arch

Surgical management of AADA demands specific consideration when AA anomalies are encountered, necessitating timely adaptation of the surgical therapeutic approach. The association of branching variants of AA sometimes does not allow the use of routine cannulation strategies. A retrospective study analyzed surgical outcomes in patients with AADA who presented with AA anomalies. The study group consisted of 896 patients with AADA who underwent surgical treatment. The researchers analyzed CT images, surgical records, and cardiopulmonary bypass records. Follow-up assessments of preoperative surviving patients were performed to record long-term mortality rates and aortic reoperation cases. Of the 81 patients with AA abnormalities, 35 had BAAs. Of all patients with AA anomalies, those with BAAs had the highest perioperative mortality (14%) and the highest incidence of neurological complications (16%) [36].

Carotid artery stenting (CAS) is a broadly utilized strategy in cases of left internal carotid artery stenosis (LICA), with good results even in high-risk surgical patients. In both T1BA and T2BA, the atypical anatomical configuration may be a more important risk factor than the stenosis itself for surgical failure and cerebral complications [48]. The achievement of CAS relies on the capacity to access the LCCA with minimal catheter manipulation and to stabilize the system throughout the procedure. Procedural safety can be ensured by establishing a tailored interventional strategy based on the identification of preprocedural BAA anatomy on CT images [49]. Choosing the best pathway to LICA for the BAA greatly simplifies the operative technique, decreasing the risk of complications during the intervention.

Montorsi et al. [37], in their study, evaluated the outcomes and success of CAS in cases of LICA stenosis in patients with BAAs at immediate, 30, and 60 days postprocedurally. Out of 505 consecutive LICA stenosis patients treated by CAS, 60 (11.9%) patients with LICA stenosis and BAAs underwent procedures employing either the right radial (n = 32) or right brachial (n = 28) artery approach. Three-quarters of the patients presented an increased interventional risk, while 87% had no neurological symptoms. Performing CAS under cerebral protection with a distal filter or with a proximal protection device performed via a transradial or transbrachial approach had a success rate of 98.3%. 96.7% of cases achieved clinical success without any adverse reactions. Clinical success without adverse events was 96.7%. Vascular complications occurred in 3.3% of patients who were part of the transbrachial approach group. A median event-free survival rate of 93% was observed. According to this study, CAS via the right transradial or transbrachial approach is a safe and successful treatment method in individuals with associated LICA stenosis and bovine AA anatomy.

One of the objectives of the study led by Burzotta et al. [38] was to investigate the impact of the BAA anatomical variant among patients receiving CAS for LICA stenosis. AA anatomy by digital subtraction angiography before CAS, intraprocedural catheter manipulation time (CMT), and unfavorable cardiac and cerebral outcomes at 30 days postprocedural for each patient were assessed. The studied cohort included 282 subjects treated by CAS using the proximal balloon occlusion or a distal neuroprotection filter. The BAA was identified in 20.5% of patients. CMT was significantly influenced by LICA in patients with bovine configuration of the AA (49.2 min in patients with BAAs vs. 37.7 min in patients with normal AA anatomy). CMT was a standalone predictor of unfavorable events at 30 days. This study suggests that atypical AA anatomies are commonly seen in CAS techniques and are correlated with longer procedural times. In addition, the longer the CMT, the higher the risk of cardiovascular and cerebral events.

CoA is a congenital heart condition characterized by a constricted aorta. The preferred surgical approach to treat CoA in infants involves surgically widening the narrowed section of the aortic by removing a portion of it and creating a larger opening. This surgery technique is typically performed on the left side of the chest and requires the placement of clamps, one near the BT and another on the descending aorta. [50]. According to the literature, the ReCoA rate after performing this procedure is approximately 4–6% [51,52]. The success of this technique is directly proportional to the size of the vessel available for aortic reconstruction. This length is determined by the clamping distance between the clamp near the BT and the CoA [23]. In the study by Turek et al. [21], the ReCoA rate was statistically significantly higher in infants with BAAs (28.6%) compared to those with normal AA (5.7%). In addition, the mean clamping index between the LSA and the maximum proximal clamp location in patients with BAAs was significantly decreased. Froud et al. [24] reported similar results in terms of significantly shorter clamping indices in infants with BAAs who underwent surgery for CoA correction by the same technique.

Primary rupture location was statistically significantly more frequent at the AA level in the group with BAAs (59.1%) compared to the group without BAAs (13.3%). Early mortality (first 24 h after surgery) was slightly higher in the group with BAAs (9.1%) compared to the group without BAAs (7.2%) but with no statistically significant difference between the two groups. In-hospital mortality was 9.1% in the group with BAAs and 14.8% in the group without BAAs. Multivariate analysis showed that the presence of a BAA is an independent risk factor for the occurrence of primary rupture in AA and for postoperative nerve damage but not for in-hospital mortality. Therefore, this study demonstrated that in AADA carousels, the BAA predicts the location of primary rupture in the AA and is an independent risk factor for postoperative neurological complications [31].

Several studies have examined the early and lasting effects in patients who underwent surgery for AD and who also had a BAA. The 157 patients were split into two groups: those with BAAs (n = 22) and those without (n = 135). Early mortality (first 24 h after surgery) was slightly higher in the cohort with BAAs (9.1%) compared to the cohort without BAAs (7.2%). The rate of in-hospital deaths was lower in patients with BAAs than in the group without this anomaly (9.1% vs. 14.8%). In addition, the presence of BAAs increased the risk of primary rupture location in AA and postoperative nerve damage, although it did not affect in-hospital mortality. Overall, this research suggests that the presence of bovine AA anatomy may be a biomarker of primary rupture site in AD and is a predictor of postoperative neurological complications.

Regarding the implications of surgical treatment of AADA in patients with BAAs, the perioperative mortality rate recorded in the cohort studied by Zhu et al. [36] is somewhat consistent with previous studies conducted by Ma et al. [53] and Waterford et al. [54] who concluded that the documented perioperative mortality rate in patients with AADA was between 5 and 15%. There is research reporting a higher frequency of neurological problems in patients with AADA [41], which is consistent with the findings of the studies included in our review that patients with AADA experienced more neurological complications and perioperative deaths compared to those with various AA abnormalities.

Zhu et al. [36] found that in patients with BAAs undergoing surgical treatment for AADA, the perioperative mortality rate aligns with rates reported in previous studies by Ma et al. [53] and Waterford et al. [54], which indicated a mortality rate of 5–15%. One study [23] showed a higher likelihood of neurological adverse events in patients with BAAs, which was supported by the findings of Zhu et al. [36], who reported higher rates of neuro-related complications and perioperative fatality rate in patients with BAAs compared to those with different other anomalies.

The CAS technique usually involves a transfemoral approach. Surgeons opt more often for this approach due to their long experience regarding the transfemoral route and due to the larger dimensions of this vessel that facilitate the mounting of certain devices [55]. When patients with carotid artery stenosis also suffer from peripheral arterial disease, performing CAS via the transfemoral route becomes difficult. From this point of view, the upper limb surgical approach, also performed in patients in the Montorsi et al. study [37], has two valuable advantages: a lower rate of significant atherosclerosis and the option to choose between the brachial and radial arteries. For the treatment of LICA stenosis, comparing the advantages of the brachial/radial approach with those of the femoral approach, there are several potential benefits: avoidance of the introduction of catheterization equipment in the AA; ensuring a smooth path to the target vessel, using tools like proximal protective devices; reduction or even absence of vascular complications and rapid recovery of locomotor function.

CAS is unanimously considered an effective therapy for treating carotid artery stenosis. At the same time, the presence of anatomical variations of AA is a key factor in predicting periprocedural outcomes. A study by Burzotta et al. [38] demonstrated that CAS can be successfully performed regardless of AA anatomy when using proximal protection or distal neuroprotection. The anatomical complexity of the BAA was found to prolong procedural time according to CMT, while adverse events at 30 days were related to increased CMT rather than complicated anatomical features.

Studies by Turek et al. [21] and Froud et al. [24] have shown there is a short anastomotic distance in the BAA, predisposing to ReCoA, previously corrected by left thoracotomy with extended head-to-head anastomosis. In addition to the short anastomosis distance, other factors may negatively impact the success rate of extended anastomosis. These include birth weight below 2.5 kg and hypoplasia of the AA arch, both of which result in reduced aortic size and clamping distance. In both cases, existing studies have shown that median sternotomy repair of CoA with cardiopulmonary bypass was associated with a decreased likelihood of ReCoA [56,57]. These findings suggest to us that CoA repair via median sternotomy with cardiopulmonary bypass may similarly improve outcomes in patients with BAAs. Presumably, this technique provides wider and more direct access to the AA, allowing the surgeon to work more precisely and efficiently in the CoA area. Because of improved access and the possibility of performing a more complex procedure, the median sternotomy approach may provide greater anastomotic stability, reducing the risk of coarctation recurrence. Easier access and a wider approach using this technique could reduce postoperative complications and improve long-term surgical outcomes.

## 4. Discussion

To homogenize the reporting and interpretation of the prevalence of BAAs, it is important to establish standardized, internationally applicable diagnostic methods. A clear definition of diagnostic criteria should also be ensured. In addition, the characteristics of the populations studied need to be taken into account. Replication of studies in different populations and geographical regions could help to confirm and understand the factors influencing the occurrence of BAAs. At the same time, in geographic areas with lower socio-economic status, improved diagnostic capacity and access to health services may lead to more accurate identification and reporting of the prevalence of anatomical variations of AA. This may involve strategies to raise awareness of the importance of screening for these abnormalities, professional training, and the development of health infrastructure in the regions concerned. The influence of hormones on AA remains an active research topic. Hormonal variations may contribute to changes in AA structure or function, but more studies would be needed to fully understand the relationship and how these gender-specific differences influence the prevalence of BAAs. Continued studies in anatomy and genetics could provide more information about the processes of AA organization and formation during fetal development and the reasons for the different prevalence of the two variants of the bovine arch.

Establishing a classification of AA branching patterns by the methods proposed by Prada et al. [11] and Karacan et al. [13] involving the use of CTA with 3D reconstruction could bring significant benefits in the field of imaging and cardiovascular medicine, contributing to a more accurate assessment and more efficient care of this category of patients. A standardized method of classifying AA branching patterns would allow communication between specialists and uniform interpretation of results in the clinical setting. To be widely implemented, this method needs to be validated in a variety of populations and clinical settings, which would give it safety, reliability, and general applicability.

Further 4D flow MRI-based studies are needed to validate the findings of Shalhub et al. [19] and to better understand the impact of changes in blood flow hemodynamics on the aortic wall caused by BAA anatomy. Short- and long-term results could be extremely useful in understanding the implications of this anatomical abnormality in increasing the risk of AD.

Adequate training and education of medical personnel and the development and implementation of standardized protocols for the evaluation of AA and adjacent structures by the US could improve the detection and correct diagnosis of the BAA and its hemodynamic implications. Correct interpretation of the results obtained by this imaging method and identification of anatomical variants should be the priorities of preoperative evaluation. We also emphasize the important role of interdisciplinary collaboration and communication between cardiologists, imaging specialists, and vascular surgeons in the evaluation of images and confirmation or exclusion of presumed anatomical abnormalities.

The results of these studies reviewed by us suggest that modifications in the branching pattern of the AA may predispose individuals to TAD. Thus, the BAA may prove to elevate the risk of TAD, which clinicians should consider when outlining a patient’s risk profile. Moreover, it is necessary to conduct a future study to validate these results before making a recommendation for monitoring and including AA abnormalities in screening guidelines for TAD. By comprehensively addressing issues such as standardization of diagnostic criteria, assessment of additional risk factors, and detailed analysis of structural and functional features and pathogenic mechanisms, we may gain a clearer and more detailed understanding of how the BAA and aortic bicuspidia contribute to the risk of TAD. This may lead to significant advances in the diagnosis accuracy and clinical care for these patients. Future research may concentrate on a detailed investigation of the mechanisms by which the BAA may determine the site of entry for AD. In addition, patients incidentally found to have a BAA may require heightened clinical surveillance.

Replication and confirmation of the observations of Syperek et al. [32] in a larger cohort regarding hemodynamic changes caused by BAA anatomy, particularly T1BA, could help explain why, in patients with embolic strokes, an embolus can form and travel to vessels even when no obvious source of embolism is found during routine post-stroke evaluations.

The findings of the study by Gold et al. [33] warrant investigation in a larger cohort of patients to establish and determine whether there is a notable variation in the occurrence of stroke between the two cerebral hemispheres depending on the branching pattern of the AA and whether the bovine arch increases the likelihood of cardioembolic stroke.

Research by Matakas et al. [34] has clarified how AA anomalies influence the occurrence of stroke on the right or left side of the brain. They found a notable tendency for emboli to affect the left hemisphere in people with a BAA, seen in about 30% of individuals. Given that left-sided strokes often have a less favorable outcome, further research is essential to determine whether patients with a BAA face a more dismal prognosis compared with those with typical anatomy. If this assumption holds, consideration of AA anatomy becomes crucial when deciding anticoagulation strategies for individuals at increased risk of cardioembolic stroke.

Given the observations of Dumfarth et al. [29] regarding the role of the BAA on stroke occurrence, studies of flow patterns in the arteries that derive from the AA are required to uncover the potential pathways leading to ischemic strokes in this category of patients.

The findings of Samadhiya et al. [35] draw attention to the need for assessment and AA during routine carotid evaluation by imaging investigations, especially in younger individuals with stroke.

It is uncertain why persons with BAAs exhibit an increased rate of adverse neurological events and death during the perioperative period relative to patients with other AA anomalies. Future research involving a larger cohort is needed to validate the impact of specific AA abnormalities on peri-surgical outcomes.

The results of the study by Montorsi P et al. [37] among an adequately sized patient group with BAAs confirm the safety and efficacy of CAS performed via the right transradial or transbrachial approach. CTA imaging assessment before performing CAS should be integrated into the diagnostic workflow to determine the optimal technique and vascular approach tailored to the vascular patient’s anatomy, enabling personalized treatment plans.

The findings of the research conducted by Burzotta et al. [38] support the idea that catheter handling during CAS procedures is a primary technical factor influencing clinical outcomes. Thorough strategizing of procedures aimed at reducing CMT would be essential to enhance the safety of the carotid stenting process.

Considering the potential for CoA recurrence in the population with BAAs, further development and refinement of the surgical techniques used is needed, including the median sternotomy approach that may be safer and more effective. In addition, to optimize immediate and extended-term consequences, techniques need to be tailored to individual patient characteristics. A more comprehensive analysis of the genetic and molecular factors implicated in the development of BAAs and CoA could provide a novel understanding of the pathophysiological process and guide new therapeutic interventions. In addition, long-term monitoring programs of patients who have received treatment could be implemented, with a focus on early detection of ReCoA and effective management of possible complications.

## 5. Conclusions

The prevalence of the BAA has significant variability across different populations, being more common in Hispanics. Additionally, it is more frequently observed in women, suggesting that genetic and hormonal factors may influence its development.

Accurate identification of the BAA using imaging techniques such as US, CTA, and MRI is crucial for planning surgical and interventional procedures. Misidentification can lead to complications during catheterization and other vascular interventions.

There is some evidence to suggest an increased risk of TAA and AD and a higher frequency of CoA and its recurrence in individuals with a BAA. This association underscores the need for vigilant monitoring and early intervention in patients with this anatomical variant.

The hemodynamic changes caused by the BAA may impact the risk of cerebrovascular events such as stroke, although more research is required to establish a definitive link.

Surgeons must account for this variant when planning procedures like CAS or surgical treatment of AADA or CoA. The anatomical differences can influence the approach and techniques used to minimize risks and improve outcomes.

Educating patients about their condition and the importance of regular follow-ups is crucial, especially for those undergoing endovascular interventional treatments.

Developing standardized clinical guidelines for the management of patients with BAA variations can help optimize care and reduce the risk of complications during diagnostic and therapeutic interventions.

## Figures and Tables

**Table 1:** Summary of included studies.

Study	Geographic Region	Imaging Modality	Number of Patients	Outcome
Moorehead PA et al., 2016 [10]	USA	CTA	817 study group: 156 patients with TAD; control group: 757 patients without TAD;	General prevalence of BAA: 31.1%; Prevalence of T1BA: 14.9%; Prevalence of T2BA: 16.2%; Statistically significantly higher prevalence of T2BA in the TAD group (23.7%) compared to controls (15.9%); Non-statistically significantly higher prevalence of T1BA in the TAD group (11.5%) compared to controls (14.9%); The prevalence of T2BA in the TAA group (24.6%) was statistically significantly higher compared to controls (15.9%); Statistically significantly higher prevalence of T2BA in the AD group (42.3%) compared to controls (30.8%). Higher statistically significant prevalence of BAA in patients with AD compared to controls (42.3% vs. 30.8%). Patients with TAD were older and had an increased prevalence of hypertension, hyperlipidemia, and aortic calcification compared with those without TAD.
Prada G et al., 2016 [11]	South America	CTA	444	Prevalence of anatomic variations of AA: 40.1%; Prevalence of different branching types of AA: Type 1 (normal branching): 59.9%; Type 2 (T1BA) “Bovine arcade” 27.9%; Type 3 (T2BA): 9.9%; Type 4 (left vertebral artery arising from AA): 2.2%; Prevalence of anatomical variations of AA by gender: women: 42.3%, men: 35.9%; The prevalence of TAD among people with AA branching variations was 14%, and it was distributed according to branching type as follows: Type 2 14.5%, Type 3 11.4%, Type 4.20%.
Karacan A et al., 2014 [13]	Turky	CTA	1000	Prevalence of normal branching types of AA: Type 1 (normal branching): 79.2%; Prevalence of anatomic variations of AA: 20.8%; Prevalence of anatomic variations of AA by gender: women:22.1%, men: 20%; General prevalence of BAA: 14.1%.
Ahn SS et al., 2014 [17]	Los Angeles, California	Angiography	90	General prevalence of BAA: 35.16%; Prevalence of T1BA: 26.88%; Prevalence of T2BA: 7.53%; Prevalence of BAA in different ethnic groups: Caucasians: 27.78%, Hispanics: 50%; Prevalence of BAA by gender: women: 40%, men: 26.67%.
Terzioğlu E et al., 2022 [18]	Turky	CTA	2037	General prevalence of BAA: 15.5%; Prevalence of BAA by gender: women: 18.2%, men: 12.8%.
Shalhub S et al., 2018 [19]	USA	4D flow MRI	552 study group: 185 patients with TBAD; control group: 367 patients without TBAD;	The prevalence of anatomical variations of AA was statistically significantly higher in patients with TBAD (40.5%) compared to controls (24.5%); The most common aortic arch branching variant was BAA (37.3% in patients with TBAD vs. 22.3% in controls), followed by aberrant SRA (2.7% in patients with TBAD vs. 0.3% in controls); Higher systolic wall shear stress along the inner curve of BAA compared with the normal AA and aberrant RSA.
Clerici G et al., 2018 [20]	Italy	US	742 including 39 patients eligible for hemodynamic evaluation: 6 patients with BAA and 33 patients with normal AA pattern	General prevalence of BAA: 6.06%; Prevalence of BAA by gender: female fetuses: 33.3%, male fetuses: 66.7%; Blood flow characteristics were similar between the BAA group and the normal AA group; There were statistically significant hemodynamic differences between the BAA group and the normal AA group.
Turek JW et al., 2018 [21]	USA	US	49	The prevalence of BAA was initially underestimated: 6.1% before the review of echocardiographic reports vs. 28.6% after the review of echocardiographic reports. The prevalence of ReCoA was statistically significantly higher in patients with BAA (28.6%) compared to patients with normal AA (5.7%); The mean anastomosis index was significantly lower in patients with BAA compared to those with normal AA.
Shaaban M et al., 2022 [22]	Saudi Arabia	CTA	700	General prevalence of BAA: 2.71%; General prevalence of CoA: 16.71%; The prevalence of BAA was statistically significantly higher in patients with CoA (5.98%) compared to those without CoA (2.06%).
Meyer AM et al., 2019 [23]	USA	CTA/Non-con-trast CT	178	General prevalence of BAA: 32.58%; The distances HV1 + HV2 and HV2 + HV3 are shorter in BAA than in normal AA in patients who underwent resection with extended end-to-end anastomosis through left thoracotomy for CoA correction.
Froud JR et al., 2020 [24]	USA	CTA/Non-con-trast CT	169	General prevalence of BAA: 34%; Both the mean clamping distance and the mean clamping index were significantly lower in BAA than in normal AA patients who underwent resection with extended end-to-end anastomosis through left thoracotomy for CoA correction.
Dumfarth J et al., 2015 [25]	USA	CT/MRI	5173 study group: 556 patients with TAD; control group: 4617 patients without TAD;	AA abnormalities were statistically significantly more frequent in patients with TAD (33.5%) compared to those in the control group (18.2%); BAA was the most common abnormal branching pattern of AA in TAD patients (24.6%), followed by isolated left vertebral artery (6.3%) and aberrant RSA (1.8%); All 3 arch variations showed a significantly higher prevalence in TAD patients compared to controls; Patients with TAD and anatomical variations of AA compared to those with TAD with normal AA had hypertension less often (73.5% vs. 81.8%) but had a higher rate of bicuspid aortic valve (40.8% vs. 30.6%); Patients with variations of AA and TAD compared to those with normal AA and TAD were significantly younger (58.6 ± 13.7 years vs. 62.4 ± 12.9 years) and required aortic arch surgery more frequently (46% vs. 34.6%).
Sun J et al., 2023 [26]	China	CT and US	449	General prevalence of BAA: 21.2%; Prevalence of BAA by gender: women: 26.3%, men: 73.7%; BAA had the highest prevalence among AA abnormalities: 79.8%; The prevalence of aortic bicuspids was statistically significantly higher in patients with BAA compared to those with normal AA: 52.6% vs. 38.1%; The diameter of the ascending aorta was greater in the BAA group than in the normal AA group, but the difference was not statistically significant; Aortic bicuspidity and male gender were predictors of TAD, but BAA was not a risk factor for TAD.
Yousef S et al., 2021 [27]	USA	CT	21,336	The prevalence of anatomical variations of AA was 2.8%; The most common AA branching pattern was BAA, with a prevalence of 58.7% of all anatomical variations of AA; The prevalence of TAA was statistically significantly higher in the group with AA anomalies compared to the group with normal AA anatomy (10.8% vs. 4.1%); Independent factors statistically significantly associated with increased risk of TAA were AA abnormalities, aortic valve pathology, male gender, and arterial hypertension.
Shang M et al., 2022 [28]	USA	CT/MRI with/without contrast	24 patients with BA și TAA 43 relatives of the 24 patients had preexisting imaging investigations available for AA anatomy evaluation.	The prevalence of BAA in relatives of patients with BAA and TAA was 53%; The heritability of BAA was very high: 0.71.
Ikeno Y et al., 2019 [29]	Japan	CT	2321 group A: 815 patients with TAD; group C: 1506 patients without TAD;	Branching abnormalities of AA were more frequent in group A patients (17.2%) compared to those in group C (14.7%); Statistically, significantly more TAA patients in group A had AA abnormalities compared to group C (20.2% vs. 14.7%), including BAA (12.3% vs. 9%) and aberrant RSA (2.6%, compared to 0.5%); Regarding TAA location, the proximal aneurysm was detected more frequently in patients with BAA (15.2%), and the distal one was detected more frequently in patients with aberrant RSA (3.7%); Regarding acute or chronic AD, no statistically significant difference in AA abnormality was found.
Dumfarth J et al., 2017 [30]	Austria, SUA	CT	315 group BAA+: 49 patients with BAA; group BAA−: 266 patients without BAA;	General prevalence of BAA in patients with AADA: 15.6%; The location of the entry site of the dissection was more frequent in AA in patients with BAA (BAA+ 46.8%) compared to those without AA abnormalities (BAA− 14.3%); Independent predictors for AA rupture were BAA and preoperative competent aortic valve; 12.4% of all patients suffered a stroke; Patients with BAA had higher stroke rates (BAA+ 24.5%) compared to those with normal AA anatomy (BAA− 10.2%); BAA emerged as an independent risk factor for stroke in the AADA.
Dumfarth J et al., 2014 [31]	Austria, SUA	CT	157 group BAA+: 22 patients with BAA; group BAA−: 135 patients without BAA;	General prevalence of BAA in patients with AADA: 14%; The location of the primary rupture was statistically significantly more frequent at the AA level in the BAA+ group (59.1%) compared to the BAA− group (13.3%); Early mortality (first 24 h after surgery) was slightly higher in the BAA+ group (9.1%) than the BAA− group (7.2%) but with no statistically significant difference between the two groups. In-hospital mortality was 9.1% in the BAA+ group and 14.8% in the BAA− group; Multivariate analysis showed that the presence of a BAA is an independent risk factor for the occurrence of primary rupture in AA and for postoperative neurologic damage but not for in-hospital mortality.
Syperek A el al., 2019 [32]	Germany	CTA	474 study group: 152 patients with stroke; control group: 322 patients without stroke;	The prevalence of BAA was statistically significantly higher in the group of patients suffering from embolic stroke compared to the control group (25.7% versus 17.1%); T1BA was identified approximately equally frequently in both groups (15.1% vs. 12.1%); T2BA was significantly higher among patients with embolic stroke than those without a history of stroke (10.5% vs. 5.0%).
Gold M et al., 2018 [33]	SUA	CT/MRI	119 group BAA+: 22 patients with BAA; group BAA−: 135 patients without BAA;	General prevalence of BAA: 33%; The most common etiologies of cardioembolic stroke were atrial fibrillation—67%, congestive heart failure with ejection fraction <30–15%; BAA patients had a 50% chance of having a left or right hemisphere stroke; No statistically significant difference in cardio-emboli stroke laterality in BAA patients was demonstrated; In patients with standard AA, there was a trend toward right hemisphere lesions, but this was not statistically significant.
Matakas JD et al., 2020 [34]	SUA	CT/MRI	615 group of patients with BAA: 191; group of patients with normal AA: 424;	Among patients with normal AA, the distribution of stroke was left in 43.6%, right in 45.1%, and bilateral in 11.3% of cases; In the group of patients with BAA, the stroke distribution was left in 51.3%, right in 35.6%, and bilateral in 13.1% of cases; 41% of BAA patients were black, and there was a statistically significant association of black race with BAA.
Samadhiya S et al., 2022 [35]	India	CT/MRI	200	Standard AA—type I (with the 3 variants, types 1, 2, 3) was the most frequent, registering a prevalence of 85.5% in the studied population; BAA (with the 3 variants, A, B, C) was the most frequent branching variation of AA: 13.5%; The age at presentation of stroke in type 1 (distance is less than 1 diameter) was 61.83 years; The age at presentation of stroke in type 2 (distance is between 1 and 2 LCCA diameters) was 59.8 years; The age at presentation of stroke in type 3 (distance is greater than 2 LCCA diameters) was 60.96 years. The age at presentation of stroke in type A (LCCA originating from BT) was 53.33 years; The age at presentation of stroke in type B (common origin of BT and LCCA) was 53.36 years; The age at presentation of stroke in type C (true BAA) was 63.25 years.
Zhu J et al., 2022 [36]	China	CT/surgical records	896	9% of patients presented abnormalities of AA, of whom 3.9% BAA; Among all patients with AA abnormalities, those with BAA had the highest perioperative mortality (14%) and the highest incidence of neurological complications (16%).
Montorsi P et al., 2014 [37]	Italy	CTA	505	11.9% of the 505 patients with LICA and BAA stenosis were treated by CAS through the right radial approach (6.4%) or right brachial approach (5.5%); CAS under cerebral protection (a distal filter or proximal MO.MA system) performed via a radial or brachial approach had a 98.3% success rate; The MO.MA system proved too short in a tall patient with a radial approach, and a filter was used; Clinical success without adverse events was 96.7% due to one retinal embolism and one minor stroke; Vascular complications occurred in 3.3% of patients in the brachial approach group; Over a mean follow-up period of 18.7, the median event-free survival rate was 93%.
Burzotta F et al., 2015 [38]	Italy	Angiography	282	Of 282 CAS, 54% were under proximal balloon occlusion and 42.2% under distal filter neuroprotection; General prevalence of BAA: 20.5%; CMT was significantly influenced by LICA in patients with BAA (49.2 min in patients with BAA vs. 37.7 min in patients with normal AA anatomy); CMT was the only independent predictor of adverse outcomes at 30 days.

BAA (bovine aortic arch), T1BA (type 1 of bovine aortic arch), T2BA (type 2 of bovine aortic arch), TAD (thoracic aortic disease), AA (aortic arch), RSA (right subclavian artery), LCCA (left common carotid artery), BT (brachiocephalic trunk), CoA (coarctation of the aorta), ReCoA (recoarctation of the aorta), TAA (thoracic aorta aneurysm), AD (thoraicic aorta dissection), TBAD (type B aorta dissection), AADA (acute aorta dissection type A), CAS (carotid artery stenting), LICA (left internal carotid artery stenosis), Mo.MA (a proximal protection device that simultaneously blocks retrograde blood flow from the external carotid artery and antegrade blood flow from the common carotid artery during CAS), CMT (catheter manipulation time), HV1 (the distance from the sinotubular junction to the midpoint of the brachiocephalic trunk in normal AA and as the distance from the sinotubular junction to the midpoint of the bovine trunk in the BAA), HV2 (the distance from the midpoint of the brachiocephalic trunk to the midpoint of the left common carotid artery in the case of normal AA, respectively 0 mm in the case of BAA), HV3 (the distance from the midpoint of the left common carotid artery to the midpoint of the LCA in the case of normal AA and the distance from the midpoint of the BT to the midpoint of the left subclavicular artery in the case of BAA).

## Data Availability

All relevant data are contained within the manuscript.

## Abbreviations

AA—aortic arch; AADA—acute DeBakey type I aortic dissection; AD—thoracic aortic dissection; BAA—bovine aortic arch; BT—brachiocephalic trunk; CAS—Carotid artery; CMT—catheter manipulation time; CoA—coarctation of the aorta; CTA—computer tomographic angiography; LCCA—left common carotid artery; LICA—left internal carotid artery stenosis; LSA—left subclavian artery; MCA—middle cerebral artery; RCCA—right common carotid artery; ReCoA—aortic recoarctation; RSA—right subclavian artery; TAA—thoracic aortic aneurysm; TAD—thoracic aortic disease; TBAD—type B aortic dissection; TPV—time to peak systolic velocity; T1BA—type I bovine aortic arch; T2BA—type II bovine aortic arch; 4D-flow MRI—four-dimensional flow magnetic resonance imaging.
